# Comparison of the Effectiveness of Three Different Combinations for Colonoscopy Preparation: A Multicenter Randomized Clinical Trial

**DOI:** 10.3390/diagnostics16020337

**Published:** 2026-01-20

**Authors:** Saša Štupar, Borut Štabuc, Bojan Tepeš, Katja Tepeš, Milan Stefanovič, Sebastian Stefanovič, Samo Plut

**Affiliations:** 1 Department of Gastroenterology, University Medical Centre Ljubljana, 1000 Ljubljana, Sloveniasamo.plut@kclj.si (S.P.); 2 Faculty of Medicine, University of Ljubljana, 1000 Ljubljana, Slovenia; 3 Diagnostic Center Rogaska, 3250 Rogaška Slatina, Slovenia; 4 Diagnostic Centre Bled, 4260 Bled, Slovenia

**Keywords:** bowel preparation, polyethylene glycol, Moviprep, Plenvu, Donat Mg

## Abstract

**Background/Objectives:** High-quality bowel preparation is essential for the diagnostic accuracy of colonoscopy, the gold standard for colorectal cancer screening. In our study, we aimed to compare the efficacy and tolerability of three bowel preparation regimens—Moviprep with Donat Mg, Plenvu, and Plenvu with Donat Mg—commonly used in clinical practice in Slovenia. **Methods**: This was a randomized, multicenter, blinded clinical trial conducted across three Slovenian gastroenterology centers. A total of 300 consecutive adult patients undergoing elective colonoscopy were randomly assigned to one of the three bowel preparation groups. Bowel cleanliness was evaluated using the Boston Bowel Preparation Scale, and lesion detection was assessed using polyp detection rate (PDR) and adenoma detection rate (ADR). Patients also completed a questionnaire assessing adverse effects, overall tolerability, and willingness to repeat the same regimen. Statistical analyses included ANOVA, chi-square, Kruskal–Wallis, and *t*-tests. **Results**: Of the 300 patients included in the final analysis, 94 received Moviprep with Donat Mg, 96 received Plenvu, and 110 received Plenvu with Donat Mg. The mean age of participants was 58.4 ± 15.6 years; 158 patients (52.7%) were male and 142 (47.3%) were female. All three regimens achieved high bowel preparation adequacy (≥95%), with no statistically significant differences in total BBPS scores, PDR, or ADR. Adverse effects were mild and comparable between groups, with thirst and bloating being the most frequently reported symptoms. Patient satisfaction and willingness to repeat the preparation were high across all regimens, with no significant differences. **Conclusions**: Moviprep with Donat Mg, Plenvu, and Plenvu with Donat Mg are all effective, safe, and well-tolerated bowel preparation regimens. All regimens exceeded ESGE minimum quality standards. While the findings suggest that each regimen is suitable for routine use, the study was not powered to establish equivalence, and regimen selection should therefore continue to consider individual patient characteristics, preferences, and clinical judgment.

## 1. Introduction

According to the Global Cancer Statistics 2022, colorectal cancer (CRC) is the second leading cause of cancer-related mortality in both sexes and ranks third in terms of incidence [1]. Colonoscopy remains the gold standard for CRC screening, as it enables the early detection of malignant and premalignant lesions. However, its diagnostic accuracy is highly dependent on the quality of bowel preparation, which ensures adequate visualization of the mucosa and potential abnormalities [2,3,4,5,6]. Inadequate bowel cleansing may result in missed lesions, prolonged procedure times, and the need for repeat colonoscopies, emphasizing the need for effective preparation regimens [2].

A variety of bowel cleansing agents are available, broadly classified into hyperosmolar solutions and iso-osmotic polyethylene glycol (PEG)-based preparations. The introduction of ascorbic acid, which has a laxative effect, has allowed for a reduction in the required PEG solution volume from 4 L to 2 L without compromising efficacy or safety [7,8,9,10,11,12].

In recent years, ultra-low-volume (<1 L PEG) formulations have been developed to further enhance patient compliance. Unlike 2 L PEG solutions, 1 L PEG formulations contain significantly higher concentrations of sodium ascorbate and ascorbic acid, as well as sodium sulfate, sodium chloride, and potassium chloride [13].

Currently, Plenvu is the only 1 L PEG bowel preparation available on the Slovenian market. Its efficacy and safety have been demonstrated in multiple clinical studies [8,14,15,16,17,18,19,20,21,22,23].

In Slovenia, the traditional approach to bowel preparation has involved 2 L PEG (Moviprep) combined with Donat Mg, a mineral water containing magnesium, magnesium sulfate, and sodium sulfate. This combination has been shown to be highly effective in achieving good to excellent bowel cleansing [24].

In our study, we aimed to compare the effectiveness of different bowel preparation protocols for colonoscopy and assess their acceptability among patients.

## 2. Materials and Methods

We conducted a multicenter prospective, randomized, blinded, three-arm clinical trial evaluating the efficacy of three bowel preparation regimens. The study was carried out at three gastroenterology centers: Diagnostic Center Bled, Diagnostic Center Rogaška, and the Department of Gastroenterology at the University Medical Center Ljubljana. It was conducted from 8 March 2023, to 31 August 2023. The study was formally registered, reviewed, and approved at the national level by the Slovenian Association of Gastroenterology and Hepatology (approval/registration number: SAGH 4/2022; date of approval: 20 October 2022).

All patients over the age of 18 who were referred to one of the three participating centers for an elective colonoscopy for diagnostic or screening purposes were invited to participate. Patients were enrolled consecutively during the study period at all participating centers. We excluded patients undergoing urgent or emergent colonoscopy and those scheduled for therapeutic colonoscopy (e.g., removal of a previously diagnosed large polyp). Additional exclusion criteria included a history of incomplete colonoscopy, known prior bowel resection, severe kidney insufficiency (GFR < 30 mL/min), heart failure (NYHA class > II), coagulation disorders, a history of severe dehydration or hypermagnesemia, bowel obstruction, delayed gastric emptying, and inability to provide informed consent.

All participants provided written informed consent prior to enrollment.

After enrollment, patients were randomly assigned in a 1:1:1 ratio to one of three bowel preparation regimens. Randomization was performed independently at each participating center using a computer-generated randomization program (Random Generator 1.1.0.0. (Microsoft Corp, Redmond, WA, USA)) to ensure balanced allocation within centers. Stratification was performed by study center. Baseline variables with potential confounding effects, including age, sex, and study center, were identified a priori based on clinical relevance and were compared between groups to assess balance following randomization.

Participants were assigned to one of the following bowel preparation protocols:

1. Moviprep (Norgine Ltd., Harefield, UK) + Donat Mg (Atlantic Droga Kolinska d.o.o., Rogaška Slatina, Slovenia) group: Patients consumed 2 L of Moviprep solution and 2 L of Donat Mg.
2. Plenvu (Norgine Ltd., Harefield, UK) group: Patients consumed 1 L of Plenvu and 1 L of clear water.
3. Plenvu + Donat Mg group: Patients consumed 1 L of Plenvu, 2 L of Donat Mg, and 1 L of clear water.

Each participant received a unique randomization number, which corresponded to a pre-prepared, sequentially numbered, opaque, sealed package containing both the bowel-preparation agent and the corresponding instructions. Packages were labeled only with the randomization code, ensuring that investigators, endoscopists and participants remained blinded to the allocated regimen. Allocation concealment was therefore maintained throughout the study, and treatment identity was disclosed only after study completion and database lock.

Colonoscopies were performed according to standard clinical practice at all participating centers. Sedation was not routinely planned and was administered only on demand in the form of conscious sedation in patients experiencing significant anxiety or discomfort during the procedure. All patients followed the same bowel preparation protocol regarding timing, with completion of bowel preparation occurring a minimum of 3 h and a maximum of 5 h before the scheduled colonoscopy. The bowel preparation regimen and timing were identical irrespective of sedation use.

During the bowel preparation phase, patients completed a questionnaire which assessed nine different categories of side effects, which were selected based on the most commonly reported adverse effects of bowel cleansing agents (Supplementary Material S1: Patient Questionnaire on Bowel Preparation Tolerability). Patients rated the severity of each side effect on a scale from 0 (none) to 4 (very severe). Additionally, the questionnaire included a question (Yes/No) on whether they would undergo the same preparation regimen again, and assessment of global tolerability score, ranging from 0 to 10 (with 10 being the best possible rating).

The primary outcomes of the study were bowel cleanliness, assessed using the Boston Bowel Preparation Scale (BBPS), polyp detection rate (PDR) and adenoma detection rate (ADR). BBPS scoring was performed according to the original validated scoring system and applied uniformly across all participating centers.

Furthermore, we wanted to evaluate the overall patient experience with bowel preparation, including the assessment of side effects, patient satisfaction with the preparation process, and willingness to use the same preparation method in the future.

The sample size was determined pragmatically, informed by sample sizes used in previous comparable studies, and based on the number of consecutive eligible patients undergoing elective colonoscopy at the three participating centers during the predefined study period.

### Statistical Analysis

Statistical analysis was performed using IBM SPSS Statistics 30.0.0.0 (IBM Corp., Armonk, NY, USA), with statistical significance set at *p* < 0.05. Normality of continuous variables was assessed using the Shapiro–Wilk test, and parametric or non-parametric tests were applied accordingly; ordinal variables were analyzed using non-parametric methods.

Patient characteristics were compared using the chi-square test, while differences in mean age were analyzed using analysis of variance (ANOVA).

ANOVA was also employed to compare bowel cleanliness, as assessed by the BBPS, the mean number of polyps among groups, and the overall tolerability score. The chi-square test was used to compare PDR and ADR between groups, as well as patients’ willingness to reuse the same bowel preparation regimen (Yes/No responses).

For pairwise comparisons of differences in BBPS scores and the mean number of polyps per colonoscopy, pairwise *t*-tests were conducted. The chi-square test was used for pairwise comparisons of PDR and ADR.

The evaluation of individual adverse effects was analyzed using the Kruskal–Wallis test, while the overall tolerability of the bowel preparation was compared using ANOVA.

## 3. Results

### 3.1. Patient Characteristics

A total of 304 patients participated in the study. In four cases, only a partial colonoscopy was performed—one patient had a history of right-sided colectomy, while three underwent incomplete procedures for reasons unrelated to bowel preparation. Patients who underwent only partial colonoscopy were excluded from the final analysis.

Thus, 300 patients were analyzed: 101 from the Clinical Department of Gastroenterology, 101 from DC Bled, and 98 from DC Rogaška. For bowel preparation, 94 patients used Moviprep with Donat Mg, 96 used Plenvu, and 110 used Plenvu with Donat Mg.

The baseline characteristics of the included patients are presented in Table 1. Statistical analysis revealed a significant difference in the distribution of sex between groups (*p* = 0.034), but this difference was no longer statistically significant after post hoc analysis with Bonferroni correction. No statistically significant differences were observed in age characteristics between groups.

### 3.2. Boston Bowel Preparation Scale

In all patients, bowel cleanliness was assessed using the BBPS. Each colon segment was evaluated separately, along with the total BBPS score. Adequate bowel cleanliness was defined as a total BBPS ≥ 6, with no individual segment scoring less than 2.

Adequate bowel cleanliness was achieved in 96.3% of cases: 97.9% in the Moviprep + Donat Mg group, 95.83% in the Plenvu group, and 95.5% in the Plenvu + Donat Mg group, with no statistically significant differences (*p* = 0.625).

Analysis of BBPS scores by individual segments and total score did not reveal any statistically significant differences among the three bowel preparation regimens. Similarly, pairwise comparisons between individual groups did not show any statistically significant differences (Table 2 and Table 3).

A lower mean score was observed for the right colon compared to the other colon segments within the same bowel preparation regimen. Statistical analysis revealed a significant difference in segmental cleanliness within the Plenvu group (*p* = 0.036) and the Plenvu + Donat Mg group (*p* = 0.027). In the Moviprep + Donat Mg group, this difference was not observed, though the results were close to statistical significance (*p* = 0.058).

### 3.3. Lesion Detection

Among the 300 colonoscopies performed, polyps were detected in 152 patients and adenomas in 140 patients.

There were no statistically significant differences in polyp or adenoma detection among the analyzed outcomes, including the mean number of polyps per colonoscopy, PDR, ADR, right colon PDR, and right colon ADR (Table 2). Pairwise comparisons between groups also did not reveal statistically significant differences (Table 3).

### 3.4. Bowel Preparation Tolerability and Experience

During bowel preparation for colonoscopy, patients completed a questionnaire assessing their cleansing experience. Adverse effects, categorized into nine groups, were rated on a 0 to 4 scale, where 0 indicated no symptoms and 4 represented a severe adverse effect (Table 4).

No statistically significant differences were observed in the frequency or severity of adverse effects among the different bowel preparation regimens. The most prominent adverse effect across all three regimens was thirst (Table 4).

**Figure 1:**
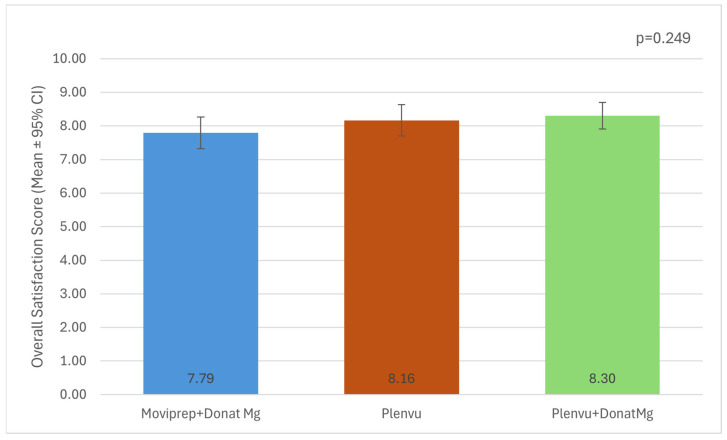
Overall satisfaction score by therapy. Bars represent mean satisfaction scores and error bars indicate 95% confidence intervals. Mean satisfaction scores were 7.79 (95% CI: 7.32–8.26) for Moviprep + Donat Mg, 8.16 (95% CI: 7.71–8.62) for Plenvu, and 8.30 (95% CI: 7.91–8.69) for Plenvu + Donat Mg. No statistically significant difference was observed between groups (ANOVA, *p* = 0.249).

Patients evaluated their overall experience with bowel preparation. The highest average rating was given to the Plenvu + Donat Mg regimen, followed by Plenvu, while the lowest rating was assigned to Moviprep + Donat Mg (Figure 1). Despite the observed differences in mean scores among the groups, statistical analysis did not reveal a significant difference (*p* = 0.249).

**Figure 2:**
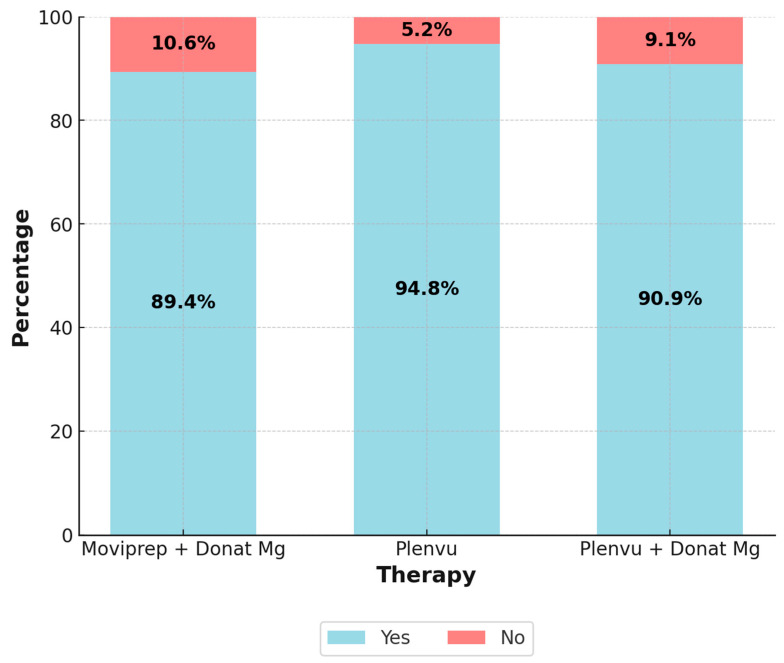
Willingness to repeat bowel preparation with the same regimen. A total of 86/96 patients (89.4%) receiving Moviprep + Donat Mg, 89/94 patients (94.8%) receiving Plenvu, and 100/110 patients (90.9%) receiving Plenvu + Donat Mg reported that they would be willing to repeat bowel preparation with the same regimen.

Patients were asked whether they would choose the same bowel preparation regimen if they required a repeat colonoscopy, the results are presented in Figure 2. Despite the minor differences between groups, statistical analysis did not reveal a significant difference (*p* = 0.375).

## 4. Discussion

Donat Mg mineral water has traditionally been used as a laxative in Slovenia [25]. Based on a study demonstrating the efficacy of Donat Mg in combination with 2 L of PEG, it is also used as a component of bowel preparation regimens for colonoscopy [24]. Several bowel preparation regimens combine PEG with osmotic, stimulant, or prokinetic agents [26,27,28,29]. To our knowledge, there was no other study using an agent similar to Donat Mg as an adjunctive component in bowel preparation.

High-quality colonoscopy is essential for the detection and removal of premalignant colorectal lesions, thereby preventing the development of CRC. The key factor in successful colonoscopy is adequate bowel preparation, as it directly impacts quality indicators such as ADR, cecal intubation rate, procedural safety, and patient tolerability [3,4,5,6,30,31,32,33,34].

Adequate bowel cleansing, as defined by the BBPS, is a total BBPS score ≥ 6 with no individual segment scoring below 2, which is considered sufficient to ensure the identification of polyps > 5 mm [35]. It is well established that the right colon more frequently harbors flat lesions, including sessile serrated lesions, which pose a greater challenge for detection [36,37,38,39]. Additionally, interval CRCs, which result from missed lesions, are more commonly found in the right colon than in the left colon [40].

A key challenge in the development of bowel preparation regimens is the effectiveness of cleansing the right colon, as its anatomical and physiological characteristics make it more difficult to clean compared to the left colon [41]. This trend was also observed in our study.

In our study, we achieved adequate bowel cleanliness in 96.3% of patients, with all three regimens achieving at least 95% adequacy. This aligns with the ESGE recommendations, which set the minimum standard for adequately prepared colonoscopies at ≥90%. Furthermore, the minimum ESGE standards for ADR (25%) and PDR (40%) were exceeded across all regimens [32].

No statistically significant differences were observed between the preparation regimens in terms of BBPS scores, both overall and by individual segments, nor in ADR and PDR for the entire colon and the right colon, which is consistent with findings from certain studies [42,43,44].

On the other hand, three larger studies contradicted our findings by demonstrating superior overall BBPS and right colon BBPS in the 1 L PEG group compared to the 2 L PEG group [19,20,21]. Similar results were reported in the CLEANSE study and in the first study conducted on an Asian population [18,22]. Additionally, Ariera et al. found that the 1 L PEG group achieved a higher overall BBPS and segmental BBPS scores compared to the 2 L PEG group. However, after excluding patients with diabetes, the differences in overall BBPS and BBPS for the transverse colon were no longer significant, while the differences for the right and left colon BBPS remained significant [17].

Similarly to BBPS scores, we did not identify any statistically significant differences in PDR, ADR, right colon PDR, right colon ADR, or the mean number of polyps per colonoscopy when comparing the preparation regimens which is similar as in two other studies [22,45]. However, a significantly higher PDR in the 1 L PEG group was observed in the national Dutch study and the Korean study [18,46]. In the MORA research group, it was found that a split-dose 1 L PEG regimen was superior to 2 L PEG in terms of higher right colon PDR, whereas this difference was not observed with a single-dose 1 L PEG regimen [19].

No statistically significant differences were observed in the frequency or severity of adverse effects among the different bowel preparation regimens. However, certain trends were noted. Thirst was the most frequently reported adverse effect across all three regimens, with the highest prevalence in the Plenvu group. Additionally, nausea was slightly more pronounced in this regimen. Bloating was more frequently reported in the Moviprep + Donat Mg and Plenvu + Donat Mg groups.

Other studies have similarly reported a higher incidence of thirst, nausea, and vomiting with 1 L PEG, which has been attributed to the higher osmolality of the preparation [15,17,18,19,20,21,47,48]. To mitigate these adverse effects, it is recommended that Plenvu be adequately chilled, consumed more slowly, and taken with a larger volume of plain water; however, these measures may reduce the efficacy of the preparation [15,19,21].

No significant differences were found in overall patient satisfaction with the bowel preparation regimen. The vast majority of study participants indicated that they would repeat the same preparation regimen if required. This result is notable, as previous studies have reported a lower willingness to repeat bowel preparation with 2 L PEG [17,44,45].

A study by Olivier et al. similarly found that over 75% of patients who had experience with both 1 L PEG and 2 L PEG preferred to repeat bowel preparation with 1 L PEG rather than 2 L PEG [49]. Brooks et al. reached similar conclusions. The lower volume of solution required for Plenvu was frequently cited in studies as the primary reason for patient preference and satisfaction [50].

Our study had several limitations. In addition to age, variability in colonoscopy indications has influenced ADR and PDR. Specific indications for colonoscopy were not recorded in our study; therefore, we cannot exclude variability between groups, which could have impacted PDR and ADR outcomes. This limitation could have been mitigated by including only patients undergoing colonoscopy within the framework of a national CRC screening program.

Additionally, we lacked data on comorbidities, which could also influence bowel cleansing effectiveness, as demonstrated in the study by Ariera et al. [17]. Although randomization ensured balance in key baseline variables, residual confounding due to unmeasured factors cannot be fully excluded. Another limitation relates to the BBPS scoring system, which, by definition, assesses bowel cleanliness after additional endoscopic cleansing. MacPhail et al. demonstrated that total BBPS scores increase by 23% following endoscopic cleansing, with the most pronounced increase observed in the right colon [51]. This finding suggests that BBPS is not an optimal indicator of the intrinsic effectiveness of a bowel preparation regimen. Since endoscopic cleansing is performed at the discretion of the endoscopist, human factors may further influence the results, potentially explaining the discrepancies in findings between different studies. Finally, the sample size was determined pragmatically without a formal power calculation; therefore, although no significant differences were observed, the study was not powered to formally establish equivalence between the regimens.

In our study, we compared the efficacy of two PEG-based preparations; however, to accurately assess regimen effectiveness and determine the potential added value of Donat Mg, an additional control group using Moviprep alone (without Donat Mg) would have been necessary.

## 5. Conclusions

In our study, none of the investigated bowel preparation regimens demonstrated superiority in terms of bowel cleanliness, PDR, or procedural tolerability. All three regimens achieved a high level of adequate preparation, exceeding the ESGE target standards.

The new low-volume PEG preparations represent a valuable alternative for patients requiring restricted fluid intake, while maintaining comparable efficacy to traditional regimens. Further research is needed to define the most optimal preparation protocols for different patient populations.

## Figures and Tables

**Table 1:** Baseline Characteristics of Patients.

Characteristics		Moviprep + Donat Mg (*n* = 94)	Plenvu (*n* = 96)	Plenvu + Donat Mg (*n* = 110)	*p*-Value
Gender	Male (*n* = 158)	45	61	52	0.034
	Female (*n* = 142)	49	35	58	
Age	Mean (SD)	57.21 (15.17)	57.21 (16.92)	60.56 (14.80)	0.20
	≤65 years (*n* = 183)	59	61	63	0.29
	>65 years (*n* = 117)	35	35	47	0.43

**Table 2:** Detailed Metrics of Results for Bowel Cleanliness Based on BBPS and Polyp Detection Across Groups. ADR—adenoma detection rate; BBPS—Boston Bowel Preparation Scale; PDR—polyp detection rate; SD—standard deviation.

	Moviprep + Donat Mg (*n* = 94)	Plenvu (*n* = 96)	Plenvu + Donat Mg (*n* = 110)	*p*-Value
BBPS				
Right colon (mean, SD)	2.65 (0.60)	2.63 (0.60)	2.65 (0.60)	0.934
Transversum (mean, SD)	2.78 (0.49)	2.80 (0.54)	2.81 (0.47)	0.838
Left colon (mean, SD)	2.82 (0.41)	2.80 (0.49)	2.81 (0.47)	0.959
Total BBPS (mean, SD)	8.24 (1.19)	8.23 (1.33)	8.29 (1.24)	0.934
Polyp detection				
Average of polyps per colonoscopy (SD)	1.57 (2.78)	1.16 (1.89)	1.08 (1.05)	0.221
PDR	51.1%	49.0%	51.2%	0.948
ADR	47.9%	44.8%	50.0%	0.756
PDR for right colon	37.2%	32.3%	39.1%	0.584
ADR for right colon	33.0%	28.1%	37.3%	0.379

**Table 3:** *p*-Values for Pairwise Comparison of BBPS and Polyp Detection. ADR—adenoma detection rate; BBPS—Boston Bowel Preparation Scale; PDR—polyp detection rate.

Outcome	Moviprep + Donat vs. Plenvu	Moviprep + Donat vs. Plenvu + Donat	Plenvu vs. Plenvu + Donat
BBPS			
Right colon	0.784	0.947	0.724
Transverse colon	0.733	0.539	0.819
Left colon	0.797	0.988	0.812
Total BBPS	0.932	0.787	0.730
Polyp Detection			
Average polyps per colonoscopy	0.264	0.120	0.693
PDR	0.772	0.982	0.780
ADR	0.670	0.762	0.455
PDR for right colon	0.474	0.786	0.310
ADR for right colon	0.467	0.522	0.164

**Table 4:** Comparison of Adverse Effects.

	Moviprep + Donat Mg (*n* = 94)	Plenvu (*n* = 96)	Plenvu + Donat Mg (*n* = 110)	*p*-Value
Sleep disturbances (mean, SD)	0.48 (1.04)	0.23 (0.75)	0.31 (0.84)	0.536
Dizziness (mean, SD)	0.44 (0.92)	0.32 (0.83)	0.18 (0.58)	0.346
Headache (mean, SD)	0.54 (1.03)	0.31 (0.80)	0.44 (0.88)	0.429
Abdominal cramps (mean, SD)	0.48 (0.95)	0.29 (0.65)	0.32 (0.86)	0.407
Bloating (mean, SD)	0.65 (1.02)	0.51 (0.91)	0.60 (1.08)	0.778
Vomiting (mean, SD)	0.23 (0.89)	0.23 (0.70)	0.30 (0.86)	0.762
Nausea (mean, SD)	0.56 (1.01)	0.68 (1.18)	0.55 (0.97)	0.941
Thirst (mean, SD)	0.91 (1.28)	1.13 (1.28)	1.01 (1.18)	0.350
Altered taste (mean, SD)	0.63 (1.04)	0.43 (0.79)	0.35 (0.79)	0.227

## Data Availability

The original contributions presented in this study are included in the article/Supplementary Material. Further inquiries can be directed to the corresponding author.

## Supplementary Materials

The following supporting information can be downloaded at: https://www.mdpi.com/article/10.3390/diagnostics16020337/s1, Supplementary Material S1: Patient Questionnaire on Bowel Preparation Tolerability.

## Abbreviations

The following abbreviations are used in this manuscript:

PEG	Polyethylene glycol
CRC	Colorectal Cancer
CI	Confidence Interval
BBPS	Boston Bowel Preparation Scale
PDR	Polyp Detection Rate
ADR	Adenoma Detection Rate
ANOVA	Analysis of Variance
NYHA	New York Heart Association
GFR	Glomerular Filtration Rate
SD	Standard Deviation
