# Supplementary material for: Comparison of the Effectiveness of Three Different Combinations for Colonoscopy Preparation: A Multicenter Randomized Clinical Trial
Source: Diagnostics (Basel). 2026 Jan 20;16(2):337. doi: 10.3390/diagnostics16020337 (PMC12840136; doi:10.3390/diagnostics16020337)
Supplement: Supplementary file 1 [file diagnostics-16-00337-s001.zip › diagnostics-3953329-supplementary.pdf]

Supplementary Material S1: Patient Questionnaire on Bowel Preparation Tolerability

Adverse effects during bowel preparation

Please evaluate the symptoms you experienced during bowel preparation for colonoscopy. For each symptom, indicate its severity using the scale below.

**Severity scale:**	**0 = None**	**1 = Mild**	**2 = Moderate**	**3 = Severe**	**4 = Very severe**
Sleep disturbances:	☐ 0	☐ 1	☐ 2	☐ 3	☐ 4
Dizziness:	☐ 0	☐ 1	☐ 2	☐ 3	☐ 4
Headache:	☐ 0	☐ 1	☐ 2	☐ 3	☐ 4
Abdominal cramps:	☐ 0	☐ 1	☐ 2	☐ 3	☐ 4
Bloating:	☐ 0	☐ 1	☐ 2	☐ 3	☐ 4
Vomiting:	☐ 0	☐ 1	☐ 2	☐ 3	☐ 4
Nausea:	☐ 0	☐ 1	☐ 2	☐ 3	☐ 4
Thirst:	☐ 0	☐ 1	☐ 2	☐ 3	☐ 4
Altered taste in the mouth:	☐ 0	☐ 1	☐ 2	☐ 3	☐ 4

Overall tolerability of the bowel preparation

Please rate your overall experience with the bowel preparation on a scale from 0 to 10:

☐ 0 (very poor) ☐ 1 ☐ 2 ☐ 3 ☐ 4 ☐ 5 ☐ 6 ☐ 7 ☐ 8 ☐ 9 ☐ 10 (excellent)

Willingness to repeat the preparation

Would you be willing to use the same bowel preparation again if you needed another colonoscopy?

☐ Yes

☐ No

Note: *This questionnaire was originally developed and administered in Slovene. The present version represents an English translation provided for transparency and reference.*
